# Fully Automated Aortic Root Localization and Tilt Alignment in Cardiac Computed Tomography

**DOI:** 10.1016/j.jscai.2025.103716

**Published:** 2025-07-23

**Authors:** Elham Mahmoudi, Vinayak Nagaraja, Mohamad Sarraf, Paul Friedman, Mohamad Alkhouli, Mackram F. Eleid, Mandeep Singh, Zachi I. Attia, Joseph D. Sobek, Mohammadreza Naderian, Fred Nugen, Bardia Khosravi, Sanaz Vahdati, Bradley J. Erickson

**Affiliations:** a Artificial Intelligence Laboratory, Department of Radiology, Mayo Clinic, Rochester, Minnesota; b Radiology Informatics Laboratory, Department of Radiology, Mayo Clinic, Rochester, Minnesota; c Department of Cardiovascular Medicine, Mayo Clinic, Rochester, Minnesota

**Keywords:** cardiac imaging, computed tomography, deep learning, transcatheter aortic valve replacement

## Abstract

**Background:**

Automated analysis of cardiac computed tomography (CCT) studies may help in personalized management and outcome prediction of patients undergoing transcatheter aortic valve replacement (TAVR). The current methods are often preceded by a manual selection of the region of interest. To address this limitation, this study aims to develop an object-oriented aortic root detection pipeline.

**Methods:**

All consecutive patients who underwent CCT for TAVR procedure, from January to July 2023 at our center, were retrospectively collected. Patients with previous prosthesis or permanent pacemaker were excluded. Baseline bounding box annotations were performed by a single expert, and tilt angle measurements were performed by 2 for interobserver comparison. A pretrained convolutional neural network was used for aortic root detection, and its performance was evaluated by recall, precision, F1, average precision at an intersection over union overlap of 50% and mean average precision (mAP) 50%-95% on 100 unseen test set. For tilt alignment, intensity thresholding, connected component, and principal component analyses were proposed. Results were evaluated by Bland-Altman comparison.

**Results:**

Of the 228 TAVR patients with preprocedural CCT, 179 were eligible, and their axial contrast-enhanced CCTs could be retrieved successfully; 100 CCTs were assigned to the test set, and the remaining to the training and validation using a 4:1 split. The model detected the aortic root with recall, precision, and F1 score of 99.0%, for all 3; mAP50 of 99.5%; and mAP50-95 of 60.4%. The tilt prediction algorithm had a mean error of 7.9° (Q1-Q3, −5.3° to 21.1°) compared with 3.3° (Q1-Q3, −6.7° to 13.4°) interobserver difference.

**Conclusions:**

This study demonstrates the robust performance of a fully automated pipeline for aortic root detection and analysis of key features in pre-TAVR CCTs. Further prospective studies are required for clinical developments.

## Introduction

Calcific aortic stenosis (AS) is the most common valvular heart disease in developed countries.^1^ Transcatheter aortic valve replacement (TAVR) is currently the standard of care for high-risk patients with significant AS,^2^^,^^3^ with comparative survival benefits across all risk cohorts. However, TAVR is associated with a higher risk of postprocedural valvular leakage and permanent pacemaker requirement than surgical valve replacement.^4^^,^^5^ Accurate preprocedural assessment of the aortic valve anatomy and its adjacent structures is crucial for making informed decisions between different management strategies and personalizing risk stratification.^6^

Pre-TAVR cardiac computed tomography (CCT) study is a cornerstone for preprocedural assessment.^7^ It reveals qualitative and quantitative information regarding the dimensions, aortic annulus, calcium distribution across the aortic valve/annulus and left ventricular outflow tract, and the relationship of the coronary arteries to the aortic annulus. However, despite the high value of manually measured anatomic landmarks, personalized periprocedural outcome prediction is still a challenge.^8^ In addition to the subjective nature of these evaluations, they are time consuming and costly. Likewise, automated analysis of the aortic root structures may improve the predictive value of CCT for procedural outcomes.

Recent advancements in deep learning have revolutionized medical imaging, enhancing the diagnosis and risk prediction of valvular heart diseases.^9^ Convolutional neural networks, capable of processing 3-dimensional (3D) imaging data, have further expanded the potential to analyze volumetric data from computed tomography scans and magnetic resonance imaging, providing a more comprehensive understanding of complex anatomical structures such as the aortic root.^10^ However, the accuracy of these models heavily depends on precise selection of the region of interest (ROI) during preprocessing. While this critical step currently relies on manual delineation by domain experts, attempts to automate ROI detection in 3D volumetric data have been challenging, with existing multistep approaches producing high accumulating errors despite good performance at individual steps.^11^

This study aimed to develop an efficient deep-learning architecture tailored for detecting and aligning of the aortic root in volumetric cardiac CCT images. An efficient timely localization of aortic root can be tailored for further optimized management decisions by the end users, as well as paving the way to personalized TAVR planning.

## Methods

### Data collection

This study was determined to be exempt from the institutional review board approval with a waiver of informed consent. All consecutive patients with native aortic valve stenosis who were candidates for TAVR and had a preprocedural chest CCT at our center, from January to July 2023, were retrospectively included in the study. Patients with a previous aortic prosthesis or permanent pacemaker were excluded. All CCT scans were performed on a Siemens SOMATOM Force CT scanner (Software Version: Syngo CT VB20A). Images were retrieved from our institute’s medical PACS in Digital Imaging and Communications in Medicine format; the axial nonconstructed images were selected and converted to NIfTI (Neuroimaging Informatics Technology Initiative) format using dcm2niix package in Python programming language.^12^ Study population characteristics, age (years), sex, number of slices, and slice thickness were extracted from images metadata and compared between training, validation, and test sets using independent-sample *t* test for continuous variables, after test of normality, and χ^2^ test for categorical variables.

An experienced imaging cardiologist (E.M.) drew 3D bounding boxes around the aortic root, limited to the sinotubular junction superiorly and the coronary cusps inferiorly, using the 3D-Slicer software (v5.2.2; Brigham and Women’s Hospital, Harvard Medical School).^13^ In addition, the acute angle of the central aortic root vector with the horizontal axis was manually measured by the angle function from the same module. The location and size of each box was recorded in a standard data format. These coordinates were converted from anatomical coordinate system to the standard image coordinate system using mathematical transformations. The source code for these transformations is available on our GitHub repository.^14^

### Model development and evaluation

For automated ROI detection in CCT images, MedYOLO, an open-source adaptation of the You Only Look Once framework for 3D volumetric medical imaging, was used.^15^^,^^16^ One hundred imaging studies were randomly assigned to the test set for internal validation, and the remaining studies were randomly assigned at a 4:1 ratio into the training and validation set for training and hyperparameter optimization, respectively. For preprocessing, the input CT scans were resized to 350 voxels and voxel intensities normalized ([voxel values + 1024]/2048), while the voxel size was not changed.

The model was trained from scratch (without any pretrained weights) on minibatches of 8 images over 1000 epochs, using an NVIDIA A100-SXM4 80GB GPU. During the inference phase, nonmaximum suppression was used with a confidence threshold of 50% to refine the predicted ROIs by filtering out similar ones. Model’s performance was assessed using the recall (also known as sensitivity), precision (also known as positive predictive value), F1 score (the harmonic mean of precision and recall), mean average precision (mAP) at intersection over union (IoU) threshold of 50% (mAP50) and the mean of mAPs between IoU thresholds of 50% and 95% (mAP50-95). Metrics were calculated based on Microsoft’s COCO (Common Objects in Context) evaluation metrics.^17^

IoU is a measure of overlapping volume between the 2 boxes, calculated as the ratio of intersection (overlapping volume) to the union (sum of the 2 volumes subtracted by the intersection volume) (Central Illustration). The mean IoU gives a general evaluation of the model’s performance, while mAP gives a more specific measure of the model’s performance at different prediction confidences by presenting the area under the precision-recall curve. mAP50 estimates model’s performance in approximate localization of ROI with at least 50% overlap and mAP50-95 estimates model’s performance in perfect alignment with an overlap as high as 95%.

**Central Illustration:**
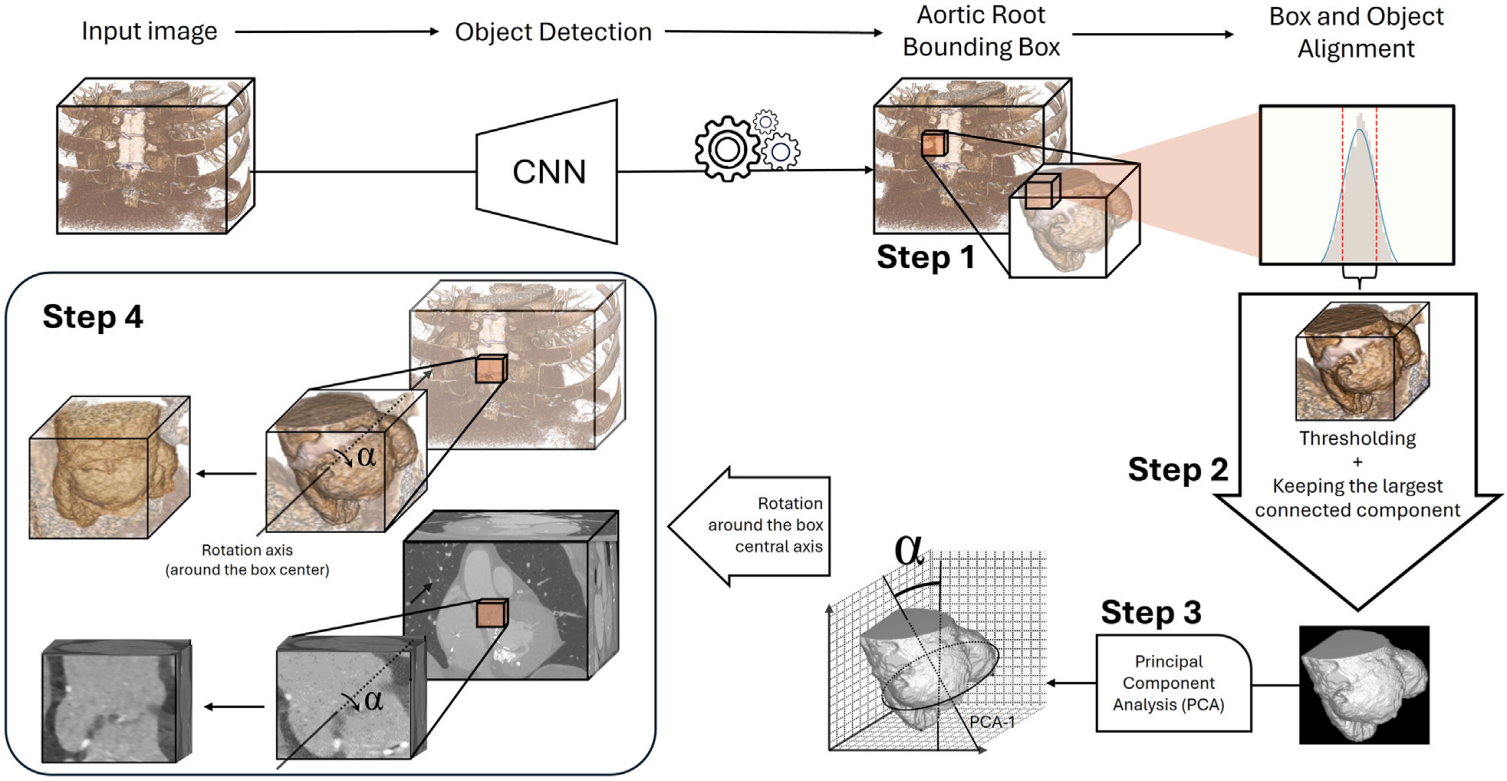
**A schematic abstract of the multistep approach to align the aortic root with the region of interest boxes.** The left ventricular outflow tract is not shown in this figure’s 3D masks, for a better understanding. CNN, convolutional neural network.

### Aortic root alignment

A multistep approach was proposed to align the detected ROI with the aortic root tilt. The goal was to automatically isolate the aortic lumen by thresholding and filter out the structures other than the contrast-enhanced luminal area by the largest connected component selection and eventually, finding the central vector of this isolated object by application of principal component analysis (PCA). The details of each step are described in schematically summarized in Central Illustration (steps 1-4). The calculated angles were then compared with manually measured angles using Bland-Altman analysis. Image processing and calculations were performed using the nibabel and numpy libraries in Python (v3.9). A second investigator independently annotated 40 images from the test set to assess interobserver reliability which was explored through using a Bland-Altman comparison.

## Results

Of the 228 contrast-enhanced thoracic aorta CCTs retrieved, 49 studies excluded for presence of prosthetic valve, permanent pacemaker, or lack of a retrievable record of axial contrast-enhanced CCTs remained. From the remaining 179 studies, random selection of 100 studies (comprising 47,250 slices) was allocated to the test set, while the remaining 79 studies were divided into 64 for training and 15 for validation sets, containing 31,495 and 7,433 slices, respectively. The mean (± SD) age of patients was 77 ± 8 years in the training set, 79 ± 7 years in the validation set, and 78 ± 8 years in the test set, with male prevalence rates of 64%, 53%, and 64%, respectively. All images had a resolution of 512 × 512 pixels, with a mean of 481 ± 109 slices per image and a mean slice thickness of 0.3 mm. Study population characteristics are summarized in Table 1.

**Table 1:** Study population characteristics

Baseline characteristic	Training (n = 64)	Validation (n = 15)	Test (n = 100)	*P*
Male sex	64%	53%	64%	.41
Age, y	77 ± 8	79 ± 7	78 ± 8	.53
Total no. of images (slice no.)	31,495	7433	47,250	—
Mean slice no. per image	492 ± 132	472 ± 95	474 ± 33	.47
Slice thickness, mm	0.3 ± <0.1	0.3 ± <0.1	0.3 ± <0.1	.67

Values are mean ± SD unless otherwise noted.

**Figure 1:**
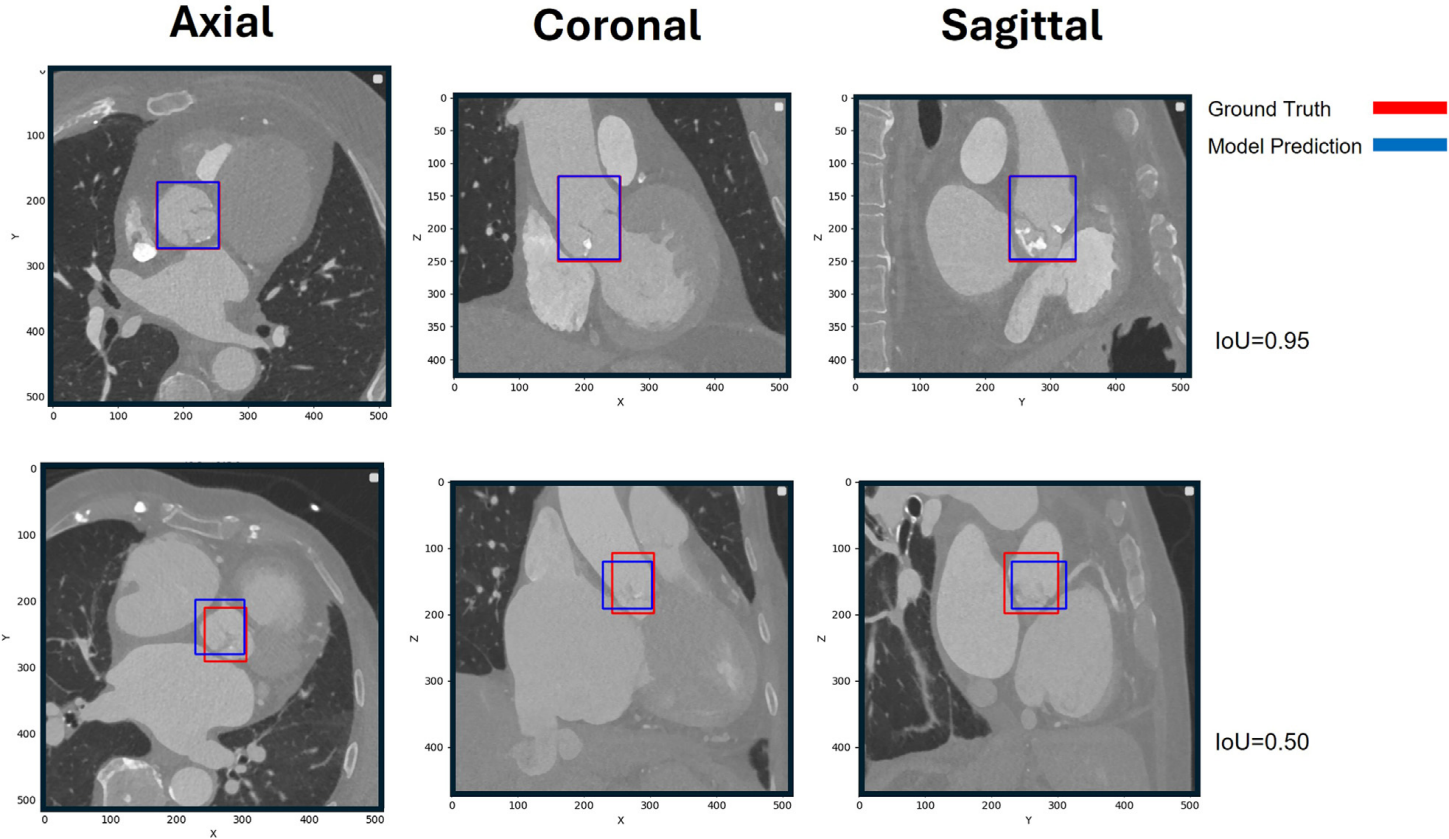
**Two samples of the test set were subject to the highest and lowest IoU between the predicted and manually annotated regions of interest.** IoU, intersection over union.

The model took 8.1 milliseconds for preprocessing, 21.8 milliseconds to detect ROIs, and 4.1 milliseconds to apply nonmaximum suppression. IoU was calculated between each pair of predicted and manual ROIs, with a minimum IoU of 50%, a mean IoU of 0.79 ± 0.08, and a maximum IoU of 95%. Recall, precision, and F1 score were 99.0%, for all 3; the mAP50 was 99.5%, and the mAP50-95 was 60.4%. Images with the highest and lowest IoUs in the test set are shown in Figure 1.

**Figure 2:**
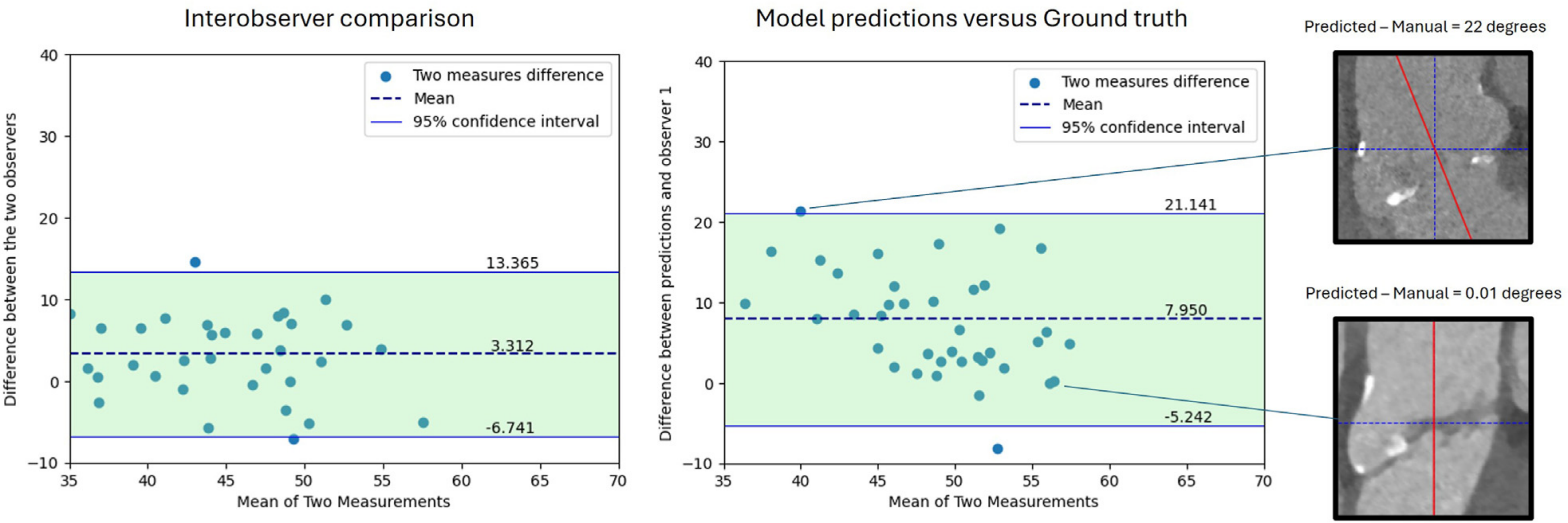
Left: Bland-Altman plot of error between two operators' measured tilt angle versus the mean value of two measurements for each image. Middle: Bland-Altman plot of error between the first observer's measurement and the actual tilt angle versus the mean value of these two angles for each image. Right: two sample images with the highest and the lowest error between the measured and actual tilt angles.

To evaluate the aortic root alignment algorithm, the thresholding and PCA algorithms were applied to the ROIs predicted by the upstream aortic root detection model. The aortic root tilt angle was measured using the proposed multistep pipeline, and the difference between automated and manual measurements was analyzed via Bland-Altman comparison. The mean aortic root tilt angle was 51.7° ± 5.5° for predicted angles and 43.7° ± 8.7° for manually measured angles. The mean deviation of predicted from the actual angles was 7.9° (Q1-Q3, −5.3° to 21.1°), while the mean difference between the 2 observers’ measurements was 3.3° (Q1-Q3, −6.7° to 13.4°). All values but 1 were in range of 95% CI. Figure 2 presents the Bland-Altman comparison of predicted versus manual measurements, interobserver differences, and images with the most and least deviations.

## Discussion

**Figure 3:**
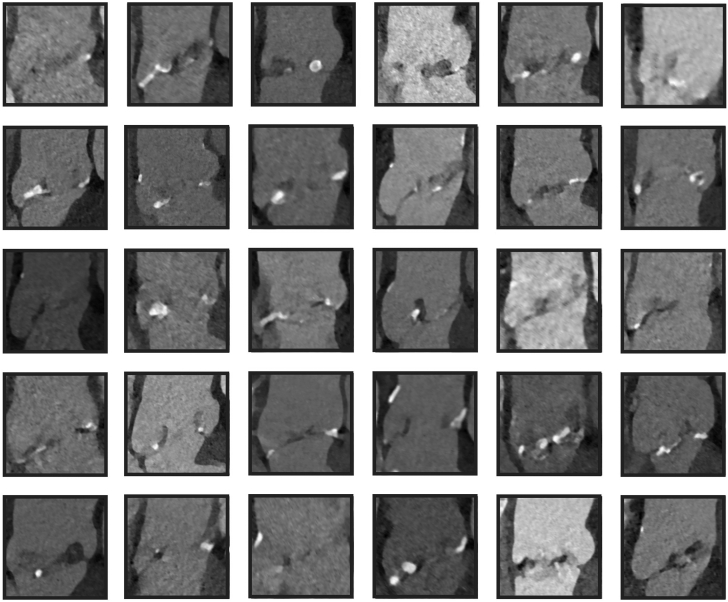
**The final result of our fully automated pipeline applied on an unseen test set.** These object-oriented 3D views of aortic root are extracted automatically from a random sample of contrast-enhanced thoracic computed tomography angiographies; the mid-coronal slice of output boxes are presented in this figure.

Our study introduces a robust object-oriented ROI detection pipeline for the aortic root region, achieving a mAP50 of 99.5% and a minimum IoU of 50%. By incorporating PCA, the detected ROIs are aligned with the aortic root’s tilt axis with a mean error of 7.9° (Q1-Q3, −5.3° to 21.1°). Figure 3 presents a random sample of ROIs detected and aligned by our method. This performance stands out among state-of-the-art methods for object detection in 3D medical imaging and the previous efforts at automated assessment of aortic root structure.^18^^,^^19^ However, a direct quantitative comparison with our results remains challenging because of the diverse approaches and evaluation metrics used in earlier studies.

### Technical considerations

The studies that implemented an initial step of ROI detection for pre-TAVR assessment are summarized in Table 2.^20^, ^21^, ^22^ Elattar et al^21^ achieved an aortic root segmentation Dice score of 95% by 4-fold subsampling of the full CCT volumes in preprocessing. Lalys et al^20^ developed a multistep pipeline to annotate important landmarks on a ROI-focused view of segmented ascending aorta. The pipeline required an initial user-defined seed point for the segmentation and later ROI detection. Isolated evaluation of these pipelines was inherently limited due to the lack of a ground-truth ROI annotation.

**Table 2:** A summary of the studies that implemented an initial step of ROI detection for pre-TAVR assessment.

Study	Year	ROI detection	Tilt alignment	ROI detection evaluation
Elattar et al^20^	2014	Automated 4-fold subsampling of the volume	No	Not applicable
Lalys et al^21^	2018	ROI selection by segmented aorta mask, coronary ostium landmarks, and user-defined seed point	Yes	Not applicable (no ground truth)
Kruger et al^22^	2022	Automated ROI selection by ROI segmentation	No	ROI-ground truth IoG of 0.92
Minghini et al^22^	2023	Automated ROI selection by ROI segmentation	No	ROI-ground truth dice score of 0.9
Saitta et al^23^	2023	Automated ROI selection based on manual segmentation and template matching	No	Not reported exclusively for the ROI detection step

IoG, intersection over ground truth; ROI, region of interest; TAVR, transcatheter aortic valve replacement.

While these methods are more cost effective, a more precise ROI delineation was possible through automated segmentation of aortic luminal area before proceeding to landmark detection and measurements. In this regard, Saitta et al^23^ used a sample of aorta segmentation masks to approximately localize aortic root on other images, which were resampled to the same size and quality as the segmented image. They reached over 90% accuracy in downstream tasks but did not report the performance of ROI detection step, exclusively. Another study by Minghini et al^24^ used an isolated segmentation of aortic root with purpose of ROI selection, leading to a dice similarity score of 90%, while Kruger et al^22^ trained a segmentation model to generate a rectangular mask on the ROI with an intersection over ground truth of 92%.

Despite the relatively good performance of these pipelines, the accuracy of the final landmark measurements was heavily dependent on the upstream segmentation task’s performance. Errors in identifying even a single landmark could lead to significant inaccuracies in more complex anatomical metrics. On the contrary, application of full-slices CCT studies was resource intensive, leading to suboptimal results in some cases, as was seen in the studies by Astudillo et al^25^ and Cho et al,^26^ which applied U-Net models to the original full-slice CT scans to annotate targeted landmarks. They observed lower than 50% dice similarity score for annular plane segmentation, pronouncing the need for limiting the input volume to selected ROIs.

Considering these limitations, Tahoces et al^11^ developed a fully automated multistep approach, using 2 classification steps for ROI sampling: an axial slice classification to isolate the thoracic rib cage and a second classification of coronal slices to select the heart box region. A 2-dimensional (2D) object detection architecture was then applied to the selected ROI’s coronal slices to identify the sections containing the aortic root. F1 score for rib cage and heart box classifications were reported as 98% and 72%, respectively, and the detected boxes had a mean distance error of 6.6 mm from the manual annotations. The aortic root tilt angle was then predicted by application of thresholding and PCA on the detected 2D ROI in the coronal view, with a mean deviation of 7.2° ± 11.4° from the manually measured angles. Despite an acceptable mean value, the measurement errors were as high as +30° or −30° in some cases, which were attributed to inaccurate upstream ROI predictions.

Our proposed approach presents a single-step 3D object detection to reduce the multiplied errors associated with multistep pipelines and led to the exceptional mAP50 of 99.5% and a tilt angle alignment with mean error of 7.9° and maximum error of 22°. Notably, our small error in tilt estimation is dominantly in a clockwise direction, which can be a result of the general clockwise rotation of the proximal ascending aorta affecting the automated estimates, while the manual measurements are merely estimated based on the aortic annular plane. This systematic deviation can be adjusted by appropriate correction methods.

This study has certain limitations. Considering the aim of the study, which was to facilitate further analysis of pre-TAVR CCTs, the training dataset included only cases of significant AS from a single center and device. Validating model’s generalizability on external datasets from other centers was limited by lack of volumetric medical imaging datasets (similar to MIMIC for 2D medical imaging) and the resource and privacy complexities of transferring large volumes of data. However, we tried to validate model’s generalizability by testing on a relatively large portion of our annotated dataset (n = 100).

### Clinical applications and future directions

In terms of pre-TAVR CCT, a precise localization of aortic root is the requisite of deep-learning models that are trained with various purposes. They may reveal hidden patterns in imaging, barely detectable by an expert radiologist. These hidden patterns help the models predict short-term and long-term postprocedural outcomes or detect a pathology. These hidden patterns are also partially trackable with some mathematical techniques to give clues on impactful structures in the predicted outcome. They can also automate the tedious process of anatomical landmark measurements required for TAVR planning, by annotating the relevant structures and landmarks including the aortic root structures, origin of the coronary arteries or the characteristics of valvular calcification. These measurements are useful in precise valve selection and implantation to reduce the risk of paravalvular leaks, valve malposition, or procedural failure. The automated pipeline of outcome prediction and landmark measurement enables time series studies on large populations to detect influencing factors on life-time prognosis and develop preventive strategies. Furthermore, traditional methods of manual landmark measurement introduce methodologic variability, which is reduced substantially by use of automated algorithms, providing clinicians with reliable and reproducible measurements that are vital during TAVR planning.

## Conclusions

This study demonstrated the robust performance of a fully automated pipeline for the detection and analysis of key features in thoracic pre-TAVR CCTs. The pipeline achieved a mAP50 of 99.5% with no IoU <50% for detecting the aortic root area. It also successfully isolated the contrast-enhanced aortic lumen and left ventricular outflow tract and predicted the aortic root tilt angle with a mean error of 7.9°, with no deviations exceeding 22°. This method can be applied to large pre-TAVR CCT samples to support the development of outcome prediction and landmark detection models, potentially improving patient outcomes by guiding TAVR teams in decision making and management planning.
